# Perception and self-assessment of digital skills and gaming among youth: A dataset from Spain

**DOI:** 10.1016/j.dib.2019.104957

**Published:** 2019-12-07

**Authors:** Daniel Aranda Juárez, Jordi Sánchez-Navarro, Leila Mohammadi

**Affiliations:** Faculty of Information and Communication Sciences, Universitat Oberta de Catalunya (UOC), Av. Tibidabo, 39-43, 08035 Barcelona, Spain

**Keywords:** Internet, Skills, Habits, Perceptions, Video games, Media, Spain, Youth

## Abstract

The present article offers a dataset of how young Spanish people perceive and evaluate their digital skills, showing the confidence level of their Social skills, Mobile skills, Information/navigation skills, Operational skills, and Creative skills. It also provides data on the use and the typology of video games in which youth are involved. This data demonstrates how young people evaluate their relationship with interactive and digital media, and supports knowledge to understand such interaction in the context of skills and abilities. It also presents socio-demographic and socio-economic characteristics, including gender, age, marital status, education, occupation and, community/residence for the Spanish population between 16 and 35 years old. This data was acquired by interviewing 1012 individuals using computer-assisted telephone interviews (CATI) in May 2017.

**Table:** Specifications Table

Subject area	Social Communication
More specific subject area	Information and Communication Technology (ICT) Skills
Type of data	SPSS, Excel, Survey and Table
How data was acquired	CATI technique: Computer-Assisted Telephone Interview
Data format	Raw and analyzed data
Experimental factors	Experimental factors data are categorized into 11 main parts which are: Socio-demographic; player-no player, the frequency of use, the most played games, money spent on games, operational skills, information and navigation skills, social skills, creative skills; mobile skills; and classification data.
Experimental features	The data are achieved through a survey in 2017, May 20–31 (N = 1012)
Data source location	Spain
Data accessibility	Data are within this article

**Table:** 

**Value of the data** • Based on an approach from the conceptualization and the framework of Helsper, van Deursen, & Eynon (2016), this dataset aims to draw a map of how the young Spanish population perceives its skills, showing the self-confidence of people in their social skills, mobile skills, information/navigation skills, operational skills and, creative skills. • The data can provide a deep insight into the Internet skills level, Internet use habits, knowledge, and social perception of the young Spanish population in the age of ubiquitous and easy access to digital devices and video games. • These data allow the evaluation and assessment of the Internet skills level and its correlation with socio-demographic and socio-economic variables such as age, gender, educational level and occupation among the Spanish population between 16 and 35 years. • These data provide an approach to the level of digital skills of youth in different dimensions. The young population is the most digital consumer, so that study of its skills is important. Present data allow establishing a starting point for relevant research that seeks to improve the level of digital literacy of youth, focusing on the skills which obtained the lowest coverage.

## 1 Data

The present dataset in this paper was collected in May 2017. The raw data are available in Excel and SPSS format. The main data file spreadsheet accompanying this article contains 1012 rows of data (representing one individual per row), with the columns containing variables derived from responses to the survey. The survey includes 15 questions followed by different possible answers located in 46 columns in the data view sheet. The survey includes 15 questions which are categorized into 11 parts: socio-demographic; player/no-player, the frequency of use, the most played games, money spent on games, operational skills, information and navigation skills, social skills, creative skills, mobile skills, and classification data.

The survey is also accompanying this article together with descriptive analysis in Table 1.

**Table 1:** Descriptive analysis results (N = 1012).

	Items	Corrected Item-Total Correlation	Cronbach's Alpha if Item Deleted	Mean	SD	Cronbach's Alpha
Operational	A) Save a photo I find online.	0.439	0.603	1.959	0.714	0.659
	B) Change the privacy settings.	0.487	0.572			
	C) Use a programming language.	0.301	0.691			
	D) Open downloaded files.	0.491	0.601			
	E) Use shortcut keys.	0.480	0.573			
Information/navigation	A) Check if information I find online is true.	0.611	0.722	1.914	0.813	0.780
	B) Choose the best keywords for online searches.	0.584	0.730			
	C) Find a website I have visited before.	0.512	0.760			
	D) Decide if I can trust a website.	0.642	0.708			
	E) Look beyond the first 3 search results.	0.465	0.768			
Social	A) Distinguish between what information you should and should not share online.	0.614	0.758	1.456	0.667	0.803
	B) Delete people from your contacts list.	0.438	0.807			
	C) Decide when you should and should not share information online.	0.699	0.729			
	D) How to behave depending on the online situation.	0.588	0.766			
	E) How to decide who to share content with.	0.621	0.759			
Creative	A) Publish videos or music you have created online.	0.492	0.784	3.148	1.106	0.795
	B) Edit or make basic changes to online content created by other people.	0.634	0.737			
	C) Differentiate between the different types of licences that apply to online content.	0.587	0.752			
	D) Design a website.	0.531	0.770			
	E) Create something new from a video or music you have found online.	0.637	0.736			
Mobile	A) Install apps on a mobile device.	0.432	0.641	1.624	0.715	0.673
	B) Monitor the costs of use of a mobile app.	0.447	0.627			
	C) Disable the “show my geographical location” function.	0.494	0.600			
	D) Block push notifications on different apps.	0.517	0.581			
	E) Take a photo or video on my smartphone and publish it on social media.	0.373	0.646			

## 2 Experimental design, materials, and methods

The presented dataset comprises raw and pre-analyzed statistical data on the internet skills level, internet use habits, internet knowledge, and social perception of the Spanish population between 16 and 35 years old, carried out in May 2017.

The Statistical Package for Social Sciences (SPSS) has been used to encode the collected data. The most significant results of the survey are detailed through the following categories: socio-demographic and socio-economic characteristics of the interviewees, gaming habits, operational skills, information/navigation skills, social skills, creative skills, and mobile skills.

Regarding the independent variables, the authors used socio-demographic characteristics which included age, gender, and marital status as well as socio-economic factors comprising education, employment, community, and residence. The participants' age was categorized into three age groups, including (16–24, 25–29, 30–35), and gender was coded as male and female. Marital status was sorted into single, married, divorced, widowed, and living with a partner. Place of residence is divided into the eighteen autonomous communities of Spain, while population size of the community were categorized into five levels (<10,000; 10,001–50,000; 50,001–100,000; 100,001–500,000; >500,000). Employment was grouped into employed, unemployed, and student, while education level was graded into seven levels.

The dependent variables presented in 9 main sections comprise Player/no-Player, the frequency of use, the most played games, money spent on games, operational skills, information and navigation skills, social skills, creative skills, and mobile skills.

The dataset has been uploaded in Figshare and it is available on: https://figshare.com/s/b816bdb62edf960aaf05.

The data has been uploaded as SPSS and Excel files while the survey is in PDF format. Readers can retrieve and reuse publicly available information by visiting the link given above.

### 2.1 Sample design and data gathering/observation methods

In order to obtain information, a structured survey has been designed. To measure Internet skills, we used the Likert-type format to provide more flexibility for interviewees, following the same scales used by Deursen, Helsper, and Eynon (2016). According to Helsper, van Deursen & Eynon (2016), various scales of self-reports are used to measure internet skills. We adapted the complete survey with some modifications in the response items in order to make them easier to understand in Spanish. According to Helsper, van Deursen & Eynon (2016), it is crucial that respondents understand the questions and answers correctly, so the response items have been changed in order to be understandable for a different language and culture.

This modification includes replacing the following items: (Not at all true of me, Not very true of me, Neither true nor untrue of me, Mostly true of me, and Very true of me) to (I know how to do this perfectly, I know how to do it, neither much nor little, I know a little how to do it, and, I don't know how to do it). Also a new option of “I don't know” has been added to the modified responses. Because “not knowing” about something could have different reasons including “Knowing about the topic but never tried” or “Not knowing anything about the topic”.

To collect the data, the Computer-Assisted Telephone Interview (CATI) technique was used for individuals aged 16–35 years living in Spain through a landline telephone. Due to having different area codes for each community of Spain, using landlines allows categorizing the data considering the autonomous communities. Moreover, it is quite cost-effective and timesaving. Considering that 78% of the households in Spain use landlines (INE, 2018, [4]), there is a high possibility to reach at least one young person through a landline.

1012 interviews were obtained. The sample was segmented by autonomous communities, proportional to the real distribution of the population, asserting the importance of considering the region of residency in the sampling of the Internet use studies. From the census, the sample was constructed based on representative quotas of the Spanish population. A public database was used with the existing landlines, and the interviews were carried out according to the established quotas.

The sampling procedure followed a three-stage selection process [1]: primary sampling units, municipalities, were randomly selected [2]; secondary sampling units, households, were randomly selected by phone number; and [3] individuals within households were randomly selected through a cross-stratification of sex and age and size of municipality which was subdivided into 7 types of communities according to their size. The survey was conducted between May 20 and May 31, 2017. The margin of error for the total sample was +3.10%, for P = Q = 50% and under the assumption of maximum indeterminacy.

There was almost an even distribution between men and women in the sample (51% of the respondents were men) with an average age of the respondents of 22 years old, and an average level of education of secondary education.

### 2.2 Social perceptions of internet skills, media, and video games

The proposed conceptualization of a range of Internet skills and social perceptions of media and video games (risks and benefits) are used to provide accurate measurement. They are as follows:

#### 2.2.1 Operational skills

The skills to operate with digital media, including opening a downloaded file and saving a photo found online, was the best-known action by respondents, while the programming language was the least known.

#### 2.2.2 Information/navigation skills

The skills to search, select, and evaluate information in digital media and the skills of navigating and orienting oneself to a hypermedia environment. Among them, finding a previously visited website and looking beyond the first three results of a search is the one best known by respondents. While to make a consultation, to check the reliability of the found information, and to make a decision of trusting a website are the least well-known information/navigation actions for them.

#### 2.2.3 Social skills

The skills to employ the information contained in digital media as a means to reach a particular, personal or professional goal. The participants showed high skills in removing people from contact lists and defining with whom to share content. To distinguish the type and the time of information to share and not share online were the two actions less known by them.

#### 2.2.4 Creative skills

The skills to create the content of acceptable quality to be published on the Internet. Creative skills are the least known by respondents. Among them, the skill to publish videos or music online was the most known action. While very few of the respondents had knowledge of how to design a website.

#### 2.2.5 Mobile skills

The different actions involved in the use of smartphone or tablets. The respondents showed high knowledge in installing applications and taking photos or videos with the smartphone while tracking the usage costs of the mobile application was the one least known by them.

#### 2.2.6 Social perceptions of the game – risks

“Video games provoke addiction” was the statement with which most respondents agreed, following by “video games cause isolation in players”. On the other hand, less agreement was shown with the statements: “they are a waste of time” and “they are violent”.

#### 2.2.7 Social perceptions of the game - benefits

“Video games stimulate memory and attention” was the statement with which most respondents agreed, followed by “Video games help develop good problem solving and strategic thinking skills.” On the other hand, less agreement was shown with “the things that are learned can be applied to daily or professional life”.

#### 2.2.8 Media and video games

When they talk about video games, the respondents believed that the media, firstly, provokes addiction, secondly it promotes violence, and finally it increases the risk of social isolation.

### 2.3 Descriptive analysis

The survey was adapted from Helsper van Deursen & Eynon (2016) and was applied to the Spanish context. The validity of the scales has been confirmed in the aforementioned study using Cronbach's alpha (α > 0.7).

The analysis of the data has been carried out using SPSS. The results showed (see Table 1) that the overall Cronbach's alpha values are relatively significant (operational & mobile ≈ 0.7) meaning that the data set is reliable. To test the consistency of the items of each scale, Table 1 provides the correlation between a particular item and the sum of the rest items (corrected item-total correlation) and the value of Cronbach's alpha if the item is deleted. For example, in the case of operational scale, item “C” has the lowest correlation (0.301) and if deleted the new alpha will become 0.691.

The statistical measures demonstrated that creative skills of Spanish people (mean = 3.148, sd = 1.106) are the most developed skills, followed by the operational skills (mean = 1.959, sd = 0.714), information skills (mean = 1.914, sd = 0.813), mobile skills (mean = 1.624, sd = 0.715), while social skills are the least developed (mean = 1.459, sd = 0.667).

## Author contributions statement

DA: Substantial contributions to the conception and design of the data set, the analysis and interpretation of data for the work. Drafting the work or revising it critically for important intellectual content.

JSN: Substantial contributions to the conception and design of the data set. Drafting.

The work or revising it critically for important intellectual content.

LM: Substantial contributions to the analysis and interpretation of data for the work.

## Ethics statements

This study was approved by the Universitat Oberta de Catalunya (Open University of Catalonia, UOC) Board of Ethics. The consent, oral and informed via telephone, was obtained from the participants before they began the survey.

The survey involves the use of anonymous information, i.e. the information never had identifiers associated with it. Our data set use of non-sensitive, completely anonymous questions. The survey and interview procedures involves participants that are not defined as “vulnerable” (according to Spanish legislation, an adult is any living participant aged 16 or over) and participation will not induce undue psychological stress or anxiety.
