# Supplementary material for: Perception and self-assessment of digital skills and gaming among youth: A dataset from Spain
Source: Data Brief. 2019 Dec 7;28:104957. doi: 10.1016/j.dib.2019.104957 (PMC6928321; doi:10.1016/j.dib.2019.104957)
Supplement: Multimedia component 1 [file mmc1.pdf]

QUESTIONNAIRE DIGITAL SKILLS

Q1a. Municipality

Less than 10,000 inhabitants	1
From 10,001 to 50,000	2
From 50,001 to 100,000	3
From 100,001 to 500,000	4
More than 500,000 inhabitants	5

Q1b. Gender. Study interviewer: enter according to the voice. If in doubt, ask: Am I speaking to a man or a woman?

Man	1
Woman	2

Q1c. How old are you?

16-24	1
25-29	2
30-35	3

Player/Non-player

1. To start with, can you tell me if you play or have played console, computer or mobile/tablet video games?

Yes	1
No	2

Frequency of use

2. On a scale of 1 to 5, where 1 = I never play, and 5 = I regularly play, could you tell me how often you play on a:

A) Console
B) Computer
C) Mobile/tablet

Quantitative 1-5	1
Doesn't know	8
Doesn’t answer	9

3. What are the games you have played most this month?

Game 1___________________________________	1
Game 2___________________________________	2
I HAVE NOT PLAYED THIS MONTH (Ignore)	3
Doesn’t know	8
Doesn’t answer	9

4. At any time, have you spent money with [GAME1 + GAME2 MENTIONED]?

Yes	1
No	2
Doesn’t know	8
Doesn’t answer	9

Filter: If Q4=1 ask

5. How much money have you spent?

Quantitative	1
Doesn’t know	8
Doesn’t answer	9

6. On a scale of 1 to 5, where 1 = occasional player, and 5 = very regular player, where would you put yourself?

Quantitative 1-5	1
Doesn’t know	8
Doesn’t answer	9

Operational skills

7. On a scale of 1 to 5, where 1 = I know exactly how to do it, and 5 = I don’t know how to do it, to what extent do you know how to perform these actions?

A) Save a photo I find online.
B) Change the privacy settings (for example, on a social media site).
C) Use a programming language (for example, Python, C +, etc).
D) Open downloaded files.
E) Use shortcut keys (for example, CTRL+C to copy, CTRL+S to save).

I know exactly how to do it	1
I know how to do it	2
I kind of know how to do it	3
I barely know how to do it	4
I don’t know how to do it	5
Doesn’t know	8
Doesn’t answer	9

Information/browsing skills

8. On a scale of 1 to 5, where 1 = I know exactly how to do it, and 5 = I don’t know how to do it, to what extent do you know how to perform these actions?

A) Check if information I find online is true.
B) Choose the best keywords for online searches.
C) Find a website I have visited before.
D) Decide if I can trust a website.
E) Look beyond the first 3 search results.

I know exactly how to do it	1
I know how to do it	2
Neither a lot nor a little	3
I barely know how to do it	4
I don’t know how to do it	5
Doesn’t know	8
Doesn’t answer	9

Social skills

9. Now, we will focus on other skills. On a scale of 1 to 5, where 1 = I know exactly how to do it, and 5 = I don’t know how to do it, to what extent do you know how to perform these actions?

A) Distinguish between what information you should and should not share online.
B) Delete people from your contacts list.
C) Decide when you should and should not share information online.
D) How to behave depending on the online situation.
E) How to decide who to share content with (for example, friends, friends of friends or everybody).

I know exactly how to do it	1
I know how to do it	2
Neither a lot nor a little	3
I barely know how to do it	4
I don’t know how to do it	5
Doesn’t know	8

Doesn't answer	9

Creative skills

10. And in terms of creative skills, to what extent do you know how to: (1 = I know exactly how to do it, and 5 = I don't know how to do it)

A) Publish videos or music you have created online.
B) Edit or make basic changes to online content created by other people.
C) Differentiate between the ~~different~~ types of licences that apply to online content.
D) Design a website.
E) Create something new from a video or music you have found online.

I know exactly how to do it	1
I know how to do it	2
Neither a lot nor a little	3
I barely know how to do it	4
I don't know how to do it	5
Doesn't know	8
Doesn't answer	9

Mobile skills

11. On a scale of 1 to 5, where 1 = I know exactly how to do it, and 5 = I don't know how to do it, to what extent do you know how to:

A) Install apps on a mobile device (for example a phone or a tablet).
B) Monitor the costs of use of a mobile app.
C) Disable the "show my geographical location" function (on Facebook, Google Maps, etc).
D) Block push notifications on different apps.
E) Take a photo or video on my smartphone and publish it on social media.

I know exactly how to do it	1
I know how to do it	2
Neither a lot nor a little	3
I barely know how to do it	4
I don't know how to do it	5
Doesn't know	8
Doesn't answer	9

S

CLASSIFICATION DATA

12. What is your employment status?

Employed	1
Disabled	2
Unemployed, having previously worked	3
Unemployed, looking for first job	4
Student	5
Housewife/Househusband	6
Doesn't answer (Ignore)	8

13. Are you employed or self-employed?

Employed	1
Self-employed	2
Doesn't answer (Ignore)	8

14. What studies have you completed?

Does not know how to read or write	1
No qualifications (incomplete primary school studies)	2
Completed primary school, approximately 10 years of age	3
Completed compulsory secondary school, until 14 years of age	4
Completed vocational training or upper secondary school, until 18 years of age	5
University diploma (3-year degree)	6
University, postgraduate or doctoral degree	7
Doesn't answer	8

15. Finally, could you tell me whether you are:

Single	1
Married	2
Living with a partner	3
Divorced/separated	4
Widow/er	6
Doesn't answer (Ignore)	8
